# Assessment of histological alterations in cartilage and extracellular matrix driven by collagen-induced arthritis in Macaca fascicularis

**DOI:** 10.1186/ar3569

**Published:** 2012-02-09

**Authors:** Norio Amizuka, Hiromi Hongo, Muneteru Sasaki, Tomoka Hasegawa, Paulo Henrique Luiz de Freitas, Hiroshi Mori, Minqi Li

**Affiliations:** 1 Dept of Develop Biol of Hard Tissue, Hokkaido University, Sapporo, Japan; 2 Dept of Oral/Maxillofacial Surg, Dr Mário Gatti Municipal Hospital, Campinas, Brazil; 3 Pharmacological Evaluation Section, Ono Pharmaceutical Co Ltd, Osaka, Japan

## Background

Arthritis is characterized by progressive cartilage erosion, inflammation of adjoining soft tissues and collapse of subchondral bone due to enhanced osteoclastic resorption. Human joints are complex structures formed by synovial tissues, articular cartilage and subchondral bone tissue. Believing on the similarities of normal joints in humans and monkeys, we have employed a model of collagen-induced arthritis in Macaca fascicularis (or crab-eating monkey) in an attempt to evaluate the histological alterations caused by such condition in the extracellular matrix of the articular cartilage.

## Materials and methods

Intermediate phalangeal proximal joints of six Macaca fascicularis suffering from collagen-induced arthritis were extracted and fixed with 4% paraformaldehyde solution. Samples were also taken from disease-free animals as controls. Tissues were embedded in paraffin or epoxy resin for histochemical and ultrastructural observations. Paraffin sections were used for alkaline phosphatase (ALP), tartrate-resistant acid phosphatase (TRAP), cathepsin K, MMP-1, type II collagen, CTX-II (fragmented type II collagen) and fibronectin staining assessments.

## Results

**Figure 1:**
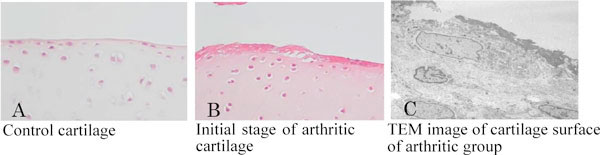


Control monkeys showed faint immunoreactivity against cathepsin K and MMP-1 in cells covering the articular cartilage and synovial tissues, indicating physiological levels of collagenous degradation. In arthritic animals, more intense cathepsin K and MMP-1 staining was observed in similar locations. ALP-positive osteoblasts and TRAP-reactive osteoclasts were abundant at the subchondral bone in arthritic samples, while control ones depicted fewer osteoclasts and weakly-stained ALP-positive osteoblasts, suggesting stimulated bone turnover in the arthritic group. Interestingly, a thick cell layer covered the articular cartilage with arthritis, and cellular debris overlaid this thick cell layer; nonetheless, articular chondrocytes seemed intact (Figure 1). In arthritic joints, the synovial tissues displayed cellular debris in abundance. CTX-II was seen in the superficial layer of the articular cartilage in arthritic samples, but it was virtually absent in the control group. Fibronectin also accumulated on the surface of the arthritic cartilage.

## Conclusion

Based on the evidence provided, it is possible that matrix degradation starts not from the adjacent subchondral bone, but from the most superficial region of the arthritic cartilage.

